# The Pharmaceutical Formulation Plays a Pivotal Role in Hydroxytyrosol Pharmacokinetics

**DOI:** 10.3390/pharmaceutics15030743

**Published:** 2023-02-23

**Authors:** Laura Di Renzo, Antonella Smeriglio, Mariarosaria Ingegneri, Paola Gualtieri, Domenico Trombetta

**Affiliations:** 1 Section of Clinical Nutrition and Nutrigenomic, Department of Biomedicine and Prevention, University of Tor Vergata, Via Montpellier 1, 00133 Rome, Italy; 2 Department of Chemical, Biological, Pharmaceutical and Environmental Sciences, University of Messina, Viale Ferdinando Stagno d’Alcontres 31, 98166 Messina, Italy

**Keywords:** hydroxytyrosol, pharmaceutical formulation, pharmacokinetics, bioavailability, DOPET, DOPAC, MOPET, HVA, human volunteers

## Abstract

Current evidence supports the use of extra virgin olive oil (EVOO) and its minor components such as hydroxytyrosol or 3,4-dihydroxyphenyl ethanol (DOPET), to improve cardiovascular and metabolic health. Nevertheless, more intervention studies in humans are needed because some gaps remain in its bioavailability and metabolism. The aim of this study was to investigate the DOPET pharmacokinetics on 20 healthy volunteers by administering a hard enteric-coated capsule containing 7.5 mg of bioactive compound conveyed in EVOO. The treatment was preceded by a washout period with a polyphenol and an alcohol-free diet. Blood and urine samples were collected at baseline and different time points, and free DOPET and metabolites, as well as sulfo- and glucuro-conjugates, were quantified by LC-DAD-ESI-MS/MS analysis. The plasma concentration versus time profiles of free DOPET was analyzed by a non-compartmental approach, and several pharmacokinetic parameters (C_max_, T_max_, T_1/2_, AUC_0–440 min_, AUC_0–∞_, AUC_t–∞,_ AUC_extrap_pred_, C_last_ and K_el_) were calculated. Results showed that DOPET C_max_ (5.5 ng/mL) was reached after 123 min (T_max_), with a T_1/2_ of 150.53 min. Comparing the data obtained with the literature, the bioavailability of this bioactive compound is about 2.5 times higher, confirming the hypothesis that the pharmaceutical formulation plays a pivotal role in the bioavailability and pharmacokinetics of hydroxytyrosol.

## 1. Introduction

The Mediterranean diet (MD) is considered a healthy and complete dietary model from a nutritional point of view [1]. Many of the beneficial effects for human health recognized and associated with this type of diet, such as longevity and the decreased incidence of chronic and inflammatory diseases [2], are due to reduced consumption of saturated fatty acids and animal proteins, a high intake of antioxidants, fibers, phytosterols, probiotics, monounsaturated fatty acids, and a correct balance of ω3/ω6 polyunsaturated fatty acids [1,3,4]. Extra virgin olive oil (EVOO), a cornerstone food of MD [5], plays a pivotal role in this dietary model thanks to its beneficial properties due to the high content of unsaturated fatty acids and phenolic compounds [6]. The main simple phenolic constituent within the EVOO is the 3,4-dihydroxy-phenylethanol (DOPET), namely also hydroxytyrosol [3,7]. Known to be the most potent antioxidant compound after gallic acid [8], hydroxytyrosol can be found in nature, mainly in olive leaves, olives, and olive oil [3]. DOPET originates from the hydrolysis of the phenolic secoiridoid oleuropein, which occurs naturally during olive ripening and olive oil production [9,10]. Indeed, the concentration of oleuropein, which is responsible for the olives’ bitter taste, progressively decreases with the fruit ripening, first transforming into its non-glycosylated form by enzymatic hydrolysis, the oleuropein aglycone, and finally into elenoic acid (non-phenolic part), and hydroxytyrosol [5,11]. Due to its amphipathic features, hydroxytyrosol can be found in free form, as acetate, or as a derivative such as oleacein, oleuropein, and verbascoside, both in olive oil and in its by-products such as pomace and olive mill wastewater [3,10].

Another natural source of this phenol is wine, although concentrations are lower than those normally found in olive oil or olive leaf extracts [3,5]. In addition to exogenous sources, DOPET can form endogenously in humans starting from dopamine [12]. Several studies have shown an increase in hydroxytyrosol biosynthesis following ethanol intake [13]. De la Torre et al. [14] compared the short-term and postprandial effects of moderate doses of EVOO and wine and found that, despite the difference in the administered doses (1.7 mg and 0.35 mg for EVOO and wine, respectively), urinary recovery of DOPET was greater after wine-coadministration, thanks to the endogenous formation of this compound from dopamine in response to alcohol intake [12].

Hydroxytyrosol shows a wide range of biological activities useful for human health [6]. Its antioxidant properties have been widely demonstrated in several in vitro and in vivo models [6], as well as in clinical studies carried out both on healthy subjects and pediatric patients affected by non-alcoholic fatty liver disease (NAFLD). In the first case, it improved body composition parameters and modulated the antioxidant profile and the expression of inflammation and oxidative stress-related genes [15], whereas, in pediatric subjects, it improved the main oxidative stress parameters, insulin resistance, and steatosis [16] as well as systemic inflammation [17]. Moreover, it has been observed that combination treatment with hydroxytyrosol and vitamin E improves NAFLD-related fibrosis [18]. Hydroxytyrosol is a powerful free radical scavenger and metal chelator, and works mainly as a chain breaker by donating a hydrogen atom to peroxyl radicals [2]. In addition to this, this compound exhibits marked anti-inflammatory, antimicrobial, antiatherogenic, and antithrombotic activities [19,20,21]. Furthermore, it has beneficial effects on endothelial dysfunction, lipids, and hemostatic profiles and can therefore be considered an effective neuroprotective, cardioprotective, and chemo-preventive compound [21]. Moreover, recently, it has been demonstrated that hydroxytyrosol could play a pivotal role in counteract long-COVID syndrome by recovering SARS-CoV-2-PLpro-dependent impairment of interferon-related genes in the polarized human airway, intestinal and liver epithelial cells [22].

Considering this, interest in hydroxytyrosol has grown a lot in recent years [20]. By the way, it has been shown that hydroxytyrosol is safe even at high doses and that it does not exhibit any genotoxicity or mutagenicity in vitro [12]. This excellent safety profile makes hydroxytyrosol an excellent candidate for nutraceutical and food industry applications [21].

All these positive aspects, however, collide with a rather lacking literature on the best formulation of this bioactive compound. Indeed, experimental studies have shown that the intestinal absorption of hydroxytyrosol is strongly influenced by the food matrix in which it is incorporated. Making a comparison between different oily and aqueous vehicles, it has been shown that when this bioactive compound is conveyed in EVOO, its bioavailability increase [20]. However, it has been recently demonstrated that both the administered DOPET and the resulting DOPET from the hydrolysis of oleuropein and other secoiridoids, main bioactive compounds within the EVOO, suffer phase II metabolism also at the gastric level, with sulphation being the main conjugation process [23]. This observation is supported by the fact that the presence of the Sulfotransferase Family 1C Member 2 (SULT1C2) isoform was detected in the stomach [24]. This could modify, even conspicuously, the amount of free DOPET available for intestinal absorption. Furthermore, to date, there are no studies available on the pharmacokinetics of this molecule in an enteric-coated pharmaceutical formulation in which DOPET is delivered in EVOO.

Based on these considerations, the aim of the present study was to evaluate, for the first time, the DOPET pharmacokinetics by administration of a new nutraceutical product consisting of enteric-coated capsules containing 7.5 mg of DOPET conveyed in EVOO to healthy volunteers.

## 2. Materials and Methods

### 2.1. Chemicals

Ethylenediaminetetraacetic Acid (EDTA), citric acid, L-ascorbic acid, β-glucuronidase type H2 from *Helix pomatia*, LC-MS grade formic acid (HCOOH), methanol, HPLC-grade (purity ≥ 97%) DOPET, 3,4-dihydroxyphenylacetic acid (DOPAC), 4-hydroxy-3-methoxyphenethanol (MOPET) and homovanillic acid (HVA) were purchased from Merck KGaA (Darmstadt, Germany).

The pharmaceutical formulation FENÒLIA^®^ enteric-coated capsules, kindly provided by P&P Farma (Turin, Italy), consists of extra virgin organic olive oil (*Olea Europaea* L., oleum ex fructibus), gelatin (shell component), coating agent: E1420, anti-caking agents: talc, silicon dioxide, dry olive extract (*Olea europaea* L., fructus) 15% titrated in DOPET, vitamin E (DL-alpha tocopheryl acetate), stabilizer: glycerol and pigment: E 171, E 141, E 161b.

### 2.2. Study Design

The study protocol, approved by the local ethics committee (Register Protocol No. 146 17/05/2018), was conducted on 20 healthy Caucasian volunteers, aged 25–60 years with BMI ranging from 19 and 25 kg/m^2^, enrolled at the University hospital facility of the Clinical Research Unit of the Department of Biomedicine and Prevention, University of Tor Vergata (Rome, Italy). Two enteric-coated capsules, each containing 7.5 mg of DOPET, were administered orally. All subjects fulfilled the following eligibility criteria: non-smokers, non-alcoholics, healthy diet, and no drugs during the experimental procedure. The administration was preceded by a 4-day washout with polyphenols and an alcoholic-free diet to avoid any interference, and by a 10-h fasting period. The study was conducted in compliance with the Declaration of Helsinki, and the selected subjects agreed to the procedure by reviewing and signing the relevant, informed consensus.

Blood samples were collected into 10 mL test tubes containing EDTA from a subcutaneous vein using a permanent catheter inserted into the forearm at baseline (T_0_) and 45, 90, 123, 150, 184, 247, 386, and 440 min. Samples were centrifuged at 1700× *g* for 10 min at 4 °C and the obtained plasma was aliquoted into test tubes containing citric acid (2 M, 10% *v*/*v*).

Urine samples were collected at baseline (T_0_) and, after the intervention, at the following mean times: 3.45, 4.18, 5.14, 6.16, 8.19, 12, and 24 h in sterile, dark polystyrene tubes (100 mL) with screw caps with 10% L-ascorbic acid as a chemical preservative. Both plasma and urine samples were immediately shipped in dry ice to the Department of Chemical, Biological, Pharmaceutical and Environmental Sciences, University of Messina (Italy) for chemical analyses, stored at −80 °C and processed within 48 h.

### 2.3. Sample Preparation

Plasma and urine samples were processed, before and after hydrolysis, according to Alemán-Jiménez et al. [20], with some modifications. Briefly, plasma and urine samples were thawed at room temperature and centrifuged (10,000× *g* for 5 min). Sample supernatants (100 and 400 μL, respectively) were hydrolyzed by incubation with 300 UI (plasma) and 1500 UI (urine) of β-glucuronidase from *Helix pomatia* for 2 h at 37 °C, clarified with 200 μL of MeOH/HCl (0.2 M) and centrifuged at 10,000× *g* for 5 min. An SPE clean-up step, by using Strata X-AW cartridges (Phenomenex, Torrance, CA, USA) mounted on VacElut Cartridge Manifolds (Agilent Technologies, Inc., Santa Clara, CA, USA), was carried out. Cartridges were conditioned and equilibrated with 2 mL of MeOH/HCOOH (98:2, *v*/*v*) and 2 mL of water/HCOOH (98:2, *v*/*v*), respectively. After sample loading, SPE cartridges were washed with water/HCOOH (98:2, *v*/*v*). Analytes were eluted with 1 mL of MeOH/HCOOH (98:2, *v*/*v*) and dried by a gentle stream of nitrogen at room temperature. Extracts were recovered with 200 μL of the mobile phase used for the LC-DAD-ESI-MS/MS analyses (see Section 2.4). Quality control samples were prepared by spiking DOPET and metabolites (DOPAC, HVA, and MOPET) in baseline control plasma and urine samples at two different concentrations (1.2 ng/mL and 5.3 ng/mL) correspondent to the limit of quantification (LOQ) in plasma and urine samples, respectively. Both precision (CV < 10%) and accuracy (≥90%) recorded in three replicates were acceptable according to ICH and FDA guidelines.

### 2.4. Quali-Quantitative Determination of DOPET and Metabolites by LC-DAD-ESI-MS/MS

Plasma and urinary DOPET and metabolites were analyzed by LC-DAD-ESI-MS/MS (Agilent Technologies, Inc., Santa Clara, CA, USA). Chromatographic analysis was carried out by a Luna Omega PS C18 column (150 mm × 2.1 mm, 5 µm; Phenomenex, Torrance, CA, USA) at 25 °C by using a mobile phase consisting of 0.1% HCOOH (Solvent A) and methanol (Solvent B) according to the following elution program: 0–18 min, 5% B; 18–21 min, 95% B; 21–30 min, 5% B; 30–35 min, 5%. The injection volume was 10 µL, and the flow rate was 0.4 mL/min. The UV–Vis spectra were recorded, ranging from 190 to 400 nm, and chromatograms were acquired at 280 nm. The experimental parameters of the mass spectrometer (ion trap, model 6320, Agilent Technologies, Santa Clara, CA, USA) equipped with an electrospray ionization interface operating in the negative (ESI−) and positive (ESI+) ionization mode were set as follows: 3.5 kV capillary voltage, 40 psi nebulizer (N2) pressure, 350 °C drying gas temperature, 9 L/min drying gas flow, and 40 V skimmer voltage. The acquisition was carried out in full-scan mode (90–1000 *m*/*z*). Mass spectra were acquired using a fragmentation energy of 1.2 V (MS/MS). Data were acquired by Agilent ChemStation software version B.01.03 and Agilent trap control software version 6.2. Quantification was carried out by building external calibration curves of commercially available reference standards (see Section 2.1).

## 3. Results

In this study, a new pharmaceutical formulation containing 7.5 mg of DOPET conveyed in EVOO was administered orally to 20 healthy volunteers. The enrolled subjects’ features are shown in Table 1.

The quali-quantitative determinations of free DOPET and metabolites were carried out by LC-DAD-ESI-MS/MS analysis (Table 2) on plasma and urine samples after oral administration of 2 cps/day, corresponding to 15 mg or 97.3 µmole of DOPET. The LC-DAD-ESI-MS/MS parameters are shown in Table 2.

**Figure 1:**
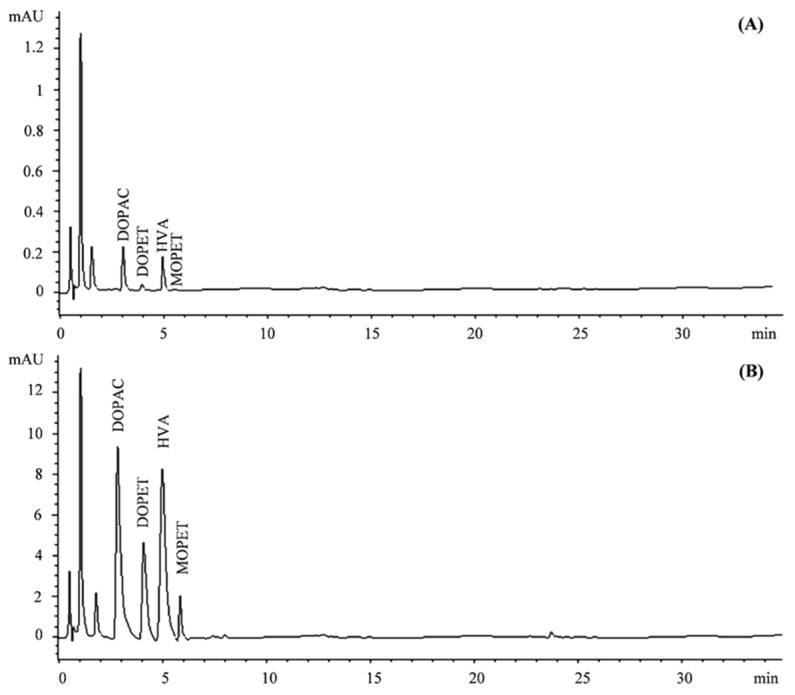
Representative chromatograms of plasma (**A**) and urine (**B**) samples acquired at 280 nm. DOPAC, 3,4-dihydroxyphenylacetic acid; DOPET, 3,4-dihydroxy-phenylethanol; homovanillic acid (HVA); 4-hydroxy-3-methoxy-phenylethanol (MOPET).

Three phase I metabolites were identified in plasma samples (T_0–440 min_): DOPAC, HVA, and MOPET. Moreover, sulfo-conjugated and glucurono-conjugated phase II derivatives have also been identified. As is possible to observe from Figure 1A,B, the chromatographic separation did not show any overlap between DOPET and metabolites, and no interference was found, at the retention times of analytes, from plasma and urine constituents.

**Figure 2:**
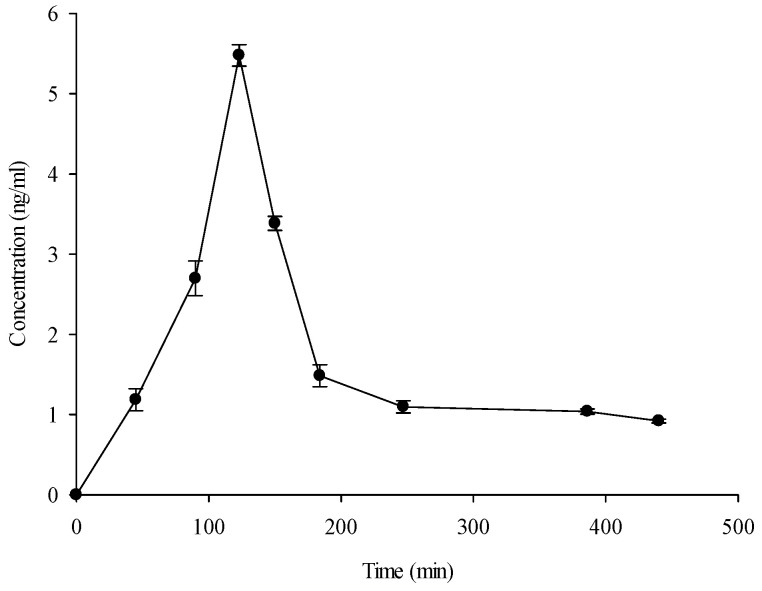
Mean plasma concentration-time profile after oral dose (15 mg) of hydroxytyrosol (3,4-dihydroxy-phenylethanol, DOPET). Results represent the concentration (ng/mL) expressed as mean ± standard deviation (*n* = 20).

The plasma concentration versus time profiles of free DOPET, following ingestion of 2 cps containing 15 mg DOPET conveyed in EVOO, was analyzed by a non-compartmental approach using Phoenix-WinNonLin software (Certara, St. Louis, MO, USA). The results are shown in Figure 2.

The mean plasma concentration-time profile shows C_max_ and T_max_ values of 5.48 ng/mL and 123 min, respectively. The T_max_ value found is compatible with a gastro-resistant formulation, considering that gastric emptying occurs in about two hours. Using the time-course values, other pharmacokinetics parameters such as half-life time (T_1/2_), the area under the curve (AUC_0–440 min_), AUC from T_0_ to T_∞_ (AUC_0–∞_), AUC extrapolated_predicted (AUC_extrap_pred_), the concentration at T_last_ (440 min) (C_last_), and first-order rate constant associated with the terminal (log-linear) portion of the curve (K_el_) were calculated (Table 3). The AUC_0–440 min_ represents the time-averaged concentration of free DOPET circulating in the plasma compartment in the time-lapse, taking into account for pharmacokinetic study.

On the contrary, AUC_0–∞_ is the AUC from time 0 extrapolated to infinite time. This parameter is calculated using the following equation:

$${\mathrm{AUC}}_{0–\infty }={\;\mathrm{AUC}}_{0–440\;}+{\;\mathrm{AUC}}_{440–\infty }$$

Assuming that DOPET is eliminated mono-exponentially after the last measurable concentration, and that no other process than elimination is involved, the terminal elimination rate of DOPET can be accurately estimated from the elimination constant calculated with the experimental data. This elimination rate is not affected by time or plasma concentrations of DOPET. Furthermore, assuming that other processes such as absorption and distribution in the terminal phase of the pharmacokinetic process are not involved, we can treat the extrapolated portion of the AUC like an IV bolus dose. Considering this, the AUC_0–440_ min can be calculated as the following:

$${\mathrm{AUC}}_{440–\infty }=\frac{C_{\mathrm{last}}}{K_{\mathrm{el}}}$$

This extrapolated AUC is then added to the observed AUC to give the total AUC value.

The small difference recorded between the AUC_0–440 min_ and AUC_0-∞_ shows that the adopted time course is enough to investigate the DOPET pharmacokinetic behavior in humans, because it highlights that, at T_440 min_, most of the free DOPET has been withdrawn from systemic circulation. For this purpose, another key parameter to calculate is the AUC_extrap_pred_ (%), the fraction of the total AUC that is due to the extrapolated AUC. Because the AUC_extrap_pred_ (%) value recorded in the present study was below 20% (mean value 18.23%), it indicates that sufficient sampling has been made for an accurate estimation of the elimination rate constant and the observed AUC.

Finally, it is possible to observe from Table 3 that, for each pharmacokinetic parameter considered, interindividual variability was recorded, although it was ≤10%.

**Figure 3:**
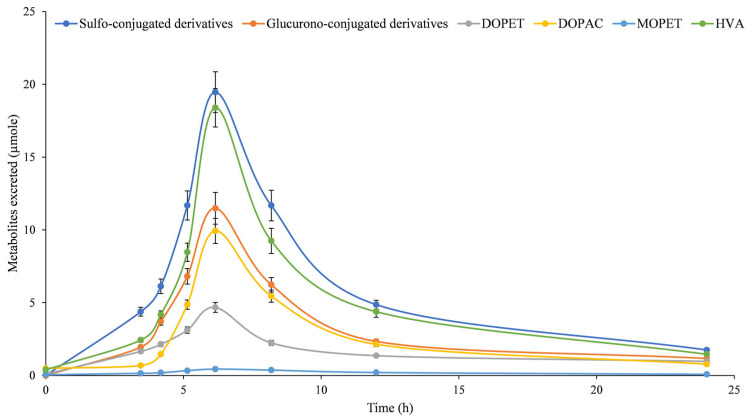
Mean concentration-time profile of metabolites present in urine after an oral dose (15 mg, 97.3 µmole) of hydroxytyrosol (3,4-dihydroxy-phenylethanol, DOPET). Results were expressed as mean amount (µmole) ± standard deviation (*n* = 20). DOPAC, 3,4-dihydroxyphenylacetic acid; DOPET, 3,4-dihydroxy-phenylethanol; homovanillic acid (HVA); 4-hydroxy-3-methoxy-phenylethanol (MOPET).

According to what has been previously made for plasma samples, also the LC-DAD-ESI-MS/MS analyses of DOPET and metabolites in urine samples were performed both before and after hydrolysis. Figure 3 shows the mean concentration (µM)-time profile of DOPET and metabolites in urine samples after a DOPET oral dose of 15 mg (97.3 µmole).

**Figure 4:**
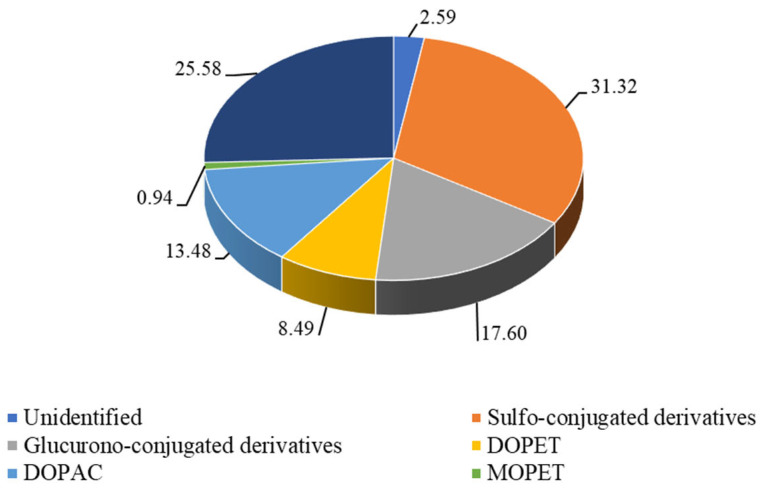
Cumulative percentage (24 h) of metabolites present in urine after oral dose (15 mg, 97.3 µmole) of hydroxytyrosol (3,4-dihydroxy-phenylethanol, DOPET). Results were expressed as mean relative area percentage (%) with respect to all identified and unidentified compounds. DOPAC, 3,4-dihydroxyphenylacetic acid; DOPET, 3,4-dihydroxy-phenylethanol; homovanillic acid (HVA); 4-hydroxy-3-methoxy-phenylethanol (MOPET).

In addition to free metabolites, sulfo-conjugated and glucurono-conjugated derivatives were also identified (Figure 3). Already from this figure, it is possible to observe as the sulfo-conjugated derivatives are the most abundant excreted metabolites followed by HVA, glucurono-conjugated derivatives, DOPAC, DOPET, and MOPET. Furthermore, it is possible to observe that the peak concentration of the parent compound and all identified metabolites, was reached approximately 6 h after DOPET administration, with 19.46, 18.39, 11.48, 9.93, 4.67, and 0.44 µmole as mean peak, respectively. Finally, expressing the cumulative results of metabolites distribution in urine (24 h) in terms of mean relative area percentage with respect to all identified and unidentified compounds (Figure 4), results do not change and, according to what mentioned above, sulfo-conjugated derivatives were found the most representative metabolites (31.32%), followed by HVA (28.58%), glucurono-conjugated derivatives (17.60%), DOPAC (13.48%), DOPET and MOPET (8.49% and 0.94%, respectively).

**Figure 5:**
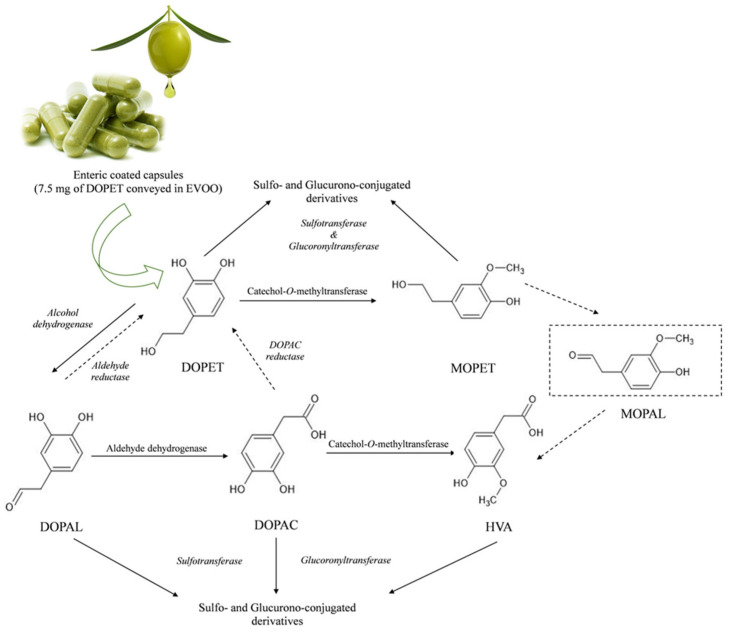
Human metabolic pathways of hydroxytyrosol (3,4-dihydroxy-phenylethanol, DOPET) conveyed in extra virgin olive oil (EVOO) and administered orally as enteric-coated capsules. 4-Hydroxy-3-methoxy-phenylethanol (MOPET); 3-methoxy-4-hydroxyphenylacetaldehyde (MOPAL); homovanillic acid (HVA); 3,4-dihydroxyphenylacetic acid (DOPAC); 3,4-Dihydroxyphenylacetaldehyde (DOPAL).

Considering the results obtained in the present study, we can therefore predict, for the investigated formulation containing DOPET conveyed in EVOO, the following metabolic pathway shown in Figure 5.

## 4. Discussion

Before discussing the pharmacokinetics of hydroxytyrosol, a premise must be made about the physical and chemical features of olive polyphenols (OP). They are structurally heterogeneous compounds with different polarities that influence their intestinal absorption. Specifically, DOPET is absorbed in the intestine by passive diffusion because of its amphiphilic properties. On the contrary, oleuropein, another abundant olive polyphenol, in its free form, is less absorbed by the enterocyte because of its hydrophobic structure and greater molecular weight. This implies that it can be degraded to DOPET [25] because of biotransformation during digestion and absorption processes, thereby raising the bioavailable content of DOPET and, in part, reaching the large intestine, where it is degraded by colonic microflora [26].

Recently, clinical trials to evaluate the pharmacokinetics of OP have increased. Accordingly, to give a clear picture in this sense, only the literature concerning human studies was discussed in the present study to identify the advantages or disadvantages deriving from a specific pharmaceutical formulation.

Gonzalez-Santiago et al. [27] analyzed the plasma concentrations of free DOPET in 10 subjects (8 males and 2 females, middle age 28 years) after an oral administration of a single dose (2.5 mg/kg b.w.) of DOPET isolated by an olive mill wastewater extract. DOPET reached the maximum plasma concentration at 13 min with a C_max_ of 1.11 μM. T_max_ and C_max_ values are compatible with those observed in the present study, considering the gastro-resistant formulation that, as such, requires about two hours from administration to make the DOPET available for absorption, and the different dose administered, about twelve times greater than that used in the present study.

Kountouri et al. [28] investigated DOPET bioavailability in 7 healthy men (middle-aged 35 years) who consumed 100 g of olives containing 76.73 mg of DOPET. Quantification of OP in plasma at different times pointed out that almost all polyphenols reached C_max_ at 1 h after olive consumption, with a plasma concentration of 3.15 µg/mL. This is the only study that reports a C_max_ so high compared to the administered dose. However, it should be emphasized that since we are dealing with 100 g of olives consumed as a single dose, and since this food is very rich in secoiridoids as well as simple phenols such as DOPET and tyrosol, this concentration could be more the result of metabolic transformations involving the more complex polyphenols which occur during gastrointestinal transit. This event, indeed, significantly increases the amount of DOPET available for absorption upstream, not to mention the effect of the food matrix, which could further facilitate the delivery of the bioactive compound. Regarding the absorption of DOPET from the food matrix, Miró-Casas et al. [29] investigated the absorption of DOPET from EVOO in 6 healthy volunteers (3 males and 3 females, middle age 36 years) showing a C_max_ and T_max_ of 25.83 ng/mL and 58 min, respectively after consumption of 25 mL of EVOO.

In another intervention study carried out on middle-aged healthy subjects (five males and four females), de Bock et al. [30] quantified the bioavailability of oleuropein and DOPET after oral administration of a pharmaceutical formulation containing an olive leaf extract as a liquid or encapsulated matrix. They showed that unlike oleuropein, the bioavailability of DOPET is not influenced by the matrix by which the polyphenols are administered, obtaining an almost overlap C_max_ (56 ng/mL vs. 59 ng/mL for the capsule and liquid matrix, respectively) and only a decrease of T_max_ of about 30 min, passing from encapsulated to the liquid formulation. Furthermore, the authors observed that HT-conjugated metabolites were the primary metabolites recovered in plasma and urine after supplementation and that gender difference in the OP bioavailability was observed.

However, recently, the influence of the food matrix on the rate of absorption and bioavailability of dietary DOPET was investigated by Alemán-Jiménez et al. [20] in a double-blind study carried out on 20 volunteers, who administered a single dose of 5 mg of DOPET through diverse food matrices: refined olive oil, flax oil, grapeseed oil, margarine, and pineapple juice. Interestingly, unlike what was stated earlier, the results revealed a strong impact of the matrices on the DOPET plasma concentration. Indeed, according to our results, while the C_max_ (3.79 ng/mL) was reached after 30 min of DOPET intake conveyed in EVOO, the intake of other matrices tested did not lead to a significant increase in the DOPET plasma concentration over time.

OP metabolic processing pathways have been extensively and deeply characterized by several animal and human studies [31]. Generally, these polyphenols undergo structural changes, mainly hydrolyzation processes by either digestive fluids in the stomach or intestines or phase I metabolic reactions [32,33] followed by phase II reactions, by which they are predominantly sulfated [32,33] or glucuronidated [29,34].

Concerning human trials, de Bock et al. [30] observed that oleuropein is extensively hydrolyzed, liberating DOPET and its aglycone and leading to an increase in the DOPET bio-accessibility. Metabolic phase II reactions cause the conjugation of DOPET, leading to a resulting high presence of sulfo- and glucuro-conjugated derivatives in plasma and urine. However, this behavior seems to be strictly dependent on the different compositions of phenolic compounds in the olive leaf extracts used in each study. Indeed, Kendall et al. [35], which carried out a study on 55 healthy young adults, who were given olive leaf supplements for 28 days, showed that neither oleuropein nor DOPET was detected in urine samples after chronic or acute consumption, suggesting that oleuropein escapes acid hydrolysis. Consequently, only oleuropein glucuronidated metabolites were identified in urine.

In confirmation of the fact that the polyphenolic composition can influence the activation of different metabolic pathways, another study carried out by Rubió et al. [33] on 12 healthy volunteers, in which EVOOs with different concentrations of phenolic content were given, showed that, after absorption, oleuropein has been extensively hydrolyzed by phase I metabolic reactions, triggering phase II metabolic reactions which have led predominantly, according to our results, to HT sulfo-conjugated. This is in accordance with García-Villalba et al. [34] and Suárez et al. [32], who revealed that oleuropein and ligstroside aglycones are hydrolyzed in the gastrointestinal tract as phase I metabolism, resulting in the polar phenols tyrosol and hydroxytyrosol. These are later conjugated by II phase metabolism and then excreted. It seems that the factor which plays a pivotal role in the activation of the metabolic reactions leading to sulfate and glucuronic acid conjugation may be the OP dosage administered [36]. Regarding OP such as oleuropein, which they are not absorbed in the small intestine, this effect is remarkable, because they reach the large intestine and, once there, is quickly transformed to DOPET by intestinal microbiota, leading to greater absorption by colon enterocytes [26]. It is well known, thanks to several animal and human studies carried out over time, that OP excretion is mainly performed via the kidneys through urine, except for compounds that escape intestinal absorption, which is directly excreted through feces per se, or after chemical transformations in the gastrointestinal tract [37]. Concerning human trials, Visioli et al. [38] investigated the presence of metabolites derived from OP in urine. For this purpose, olive oils enriched with four different phenolic extracts (20–84 μg/mL of DOPET and 36–140 μg/mL of tyrosol, respectively) was administered to 6 healthy male volunteers. Results expressed as a percentage of urine excretion with respect to the dose administered showed 29–40% for DOPET and 21–24% for tyrosol in 24 h [38]. Furthermore, Khymenets et al. [39], investigating the excretion rates of phenols and their conjugates in 24 h urine after a single dose of 50 mL of EVOO, observed the same trend with the maximum recovery of olive polyphenols’ metabolites, according to our results, in 6 h urine samples. Alemán-Jiménez et al. [20] also quantified the free DOPET and relative metabolites in urine samples at 24 h after treatment with a single dose of 5 mg of DOPET through diverse food matrices in 20 subjects. Once again, the DOPET intake by its natural source (EVOO), showed, according to our results, significantly higher urinary levels of DOPET compared to basal urine, whereas DOPET metabolites did not show any significant changes depending on the matrix administered. Finally, confirming once again our results, no gender differences were found. These results were also confirmed by Khymenets et al. [40], who showed that urine levels of DOPET and its metabolites, after supplementation with 5 or 25 mg of DOPET/day for one week, accounted for 21 and 28% of the DOPET administered, respectively. Furthermore, according to our results, the predominant forms of DOPET excreted in urine were sulfo-conjugated (16.88–23.36%), followed by glucurono-conjugated (4.70–5.01%) and free DOPET form (0–0.02%). Finally, regarding the use of a pharmaceutical formulation, also de Bock et al. [30] confirmed what was previously observed evaluating the excretion of DOPET metabolites after the administration of an olive leaf extract. Indeed, the analysis of the DOPET’s urinary metabolites revealed the predominance of sulfo- and glucurono-conjugates, whose concentration increases in the first 8 h after ingestion.

## 5. Conclusions

In conclusion, what emerges from the present and previous human studies is that, in addition to the dose of treatment and nutraceutical matrix (synthetic hydroxytyrosol, leaf extract, or olive fruit extract), another critical aspect that should be considered when designing a new pharmaceutical formulation is the vehicle within the DOPET is conveyed.

According to our results, the EVOO is the best vehicle, which leads to the major absorption of DOPET, ensuring greater bioavailability, about 2.5 times greater with respect to previous results at the same dose administered. Furthermore, in this regard, our study carried out on a greater number of subjects (20 vs. 7–10 subjects) confirms a negligible individual and gender variability in the DOPET bioavailability.

## Figures and Tables

**Table 1:** Features of enrolled healthy subjects.

Parameters	Values
Participants	20
Weight (kg)	65.1 ± 2.4
Height (cm)	169.2 ± 4.0
BMI (kg/m^2^)	22.8 ± 1.0
Age (years)	49.6 ± 5.9
Sex (M/F)	9/11

Values were expressed as mean ± standard deviation (*n* = 20) for continuous variables. Abbreviations: Body Mass Index (BMI).

**Table 2:** LC-DAD-ESI-MS/MS parameters for the quali-quantitative determination of hydroxytyrosol (3,4-dihydroxy-phenylethanol, DOPET), 3,4-dihydroxyphenylacetic acid (DOPAC), 4-Hydroxy-3-methoxyphenethanol (MOPET) and homovanillic acid (HVA) in plasma and urine samples.

Analyte	RT (min)	ESI Mode	[M-H]^−^/[M-H]^+^ (*m*/*z*)	MS/MS (*m*/*z*)	λ_max_ (nm)
DOPAC	3.263	Positive	169/	123	280
DOPET	4.042	Negative	153/	123	280
HVA	5.054	Positive	/183	137	280
MOPET	5.431	Positive	/169	151	280

**Table 3:** Pharmacokinetic parameters (mean ± standard deviation) in humans following oral administration of 15 mg of hydroxytyrosol (3,4-dihydroxy-phenylethanol, DOPET) (*n* = 20).

Formulation	Subject	T_1/2_ (min)	T_max_ (min)	C_max_ (ng/mL)	AUC_0–t_ (min*ng/mL)	AUC_0–∞_ (min*ng/mL)	AUC_t–∞_ (min*ng/mL)	AUC_extrap_pred_ (%)	C_last_ (ng/mL)	K_el_ (1/min)
Cps	1	149.933	123.000	5.294	712.679	892.913	157.173	19.306	0.761	0.005
	2	150.601	123.000	5.339	723.268	890.490	140.285	18.416	0.758	0.005
	3	148.323	123.000	5.478	695.670	880.506	149.079	17.557	0.732	0.005
	4	147.841	123.000	5.455	747.088	884.379	153.415	17.838	0.741	0.004
	5	149.380	123.000	5.398	706.174	902.077	154.248	17.937	0.744	0.005
	6	148.145	123.000	5.488	723.391	881.723	186.829	17.663	0.766	0.005
	7	151.791	123.000	5.610	776.967	902.792	158.029	17.517	0.730	0.005
	8	149.106	123.000	5.636	721.224	883.552	169.367	17.964	0.750	0.004
	9	151.339	123.000	5.547	744.207	877.017	157.389	17.496	0.761	0.005
	10	151.423	123.000	5.437	693.288	928.467	155.505	18.052	0.713	0.005
	11	150.435	123.000	5.492	693.742	889.982	173.295	18.456	0.734	0.005
	12	147.188	123.000	5.319	687.641	900.942	149.048	17.905	0.780	0.005
	13	145.501	123.000	5.540	730.696	869.484	124.409	18.387	0.731	0.005
	14	151.564	123.000	5.388	785.410	906.364	147.764	18.551	0.693	0.005
	15	147.207	123.000	5.650	723.804	892.232	181.822	18.185	0.751	0.005
	16	150.346	123.000	5.640	714.127	930.630	159.252	19.351	0.769	0.005
	17	147.919	123.000	5.502	716.517	907.057	180.365	18.262	0.735	0.005
	18	150.308	123.000	5.560	702.227	908.825	163.785	18.656	0.712	0.005
	19	152.516	123.000	5.548	728.582	881.112	155.566	18.511	0.748	0.005
	20	143.372	123.000	5.531	732.654	918.608	184.160	18.595	0.742	0.005
	N	20	20	20	20	20	20	20	20	20
	Mean	149.212	123.000	5.493	722.968	896.457	160.039	18.230	0.743	0.005
	SD	2.299	0.000	0.106	25.857	16.814	15.649	0.528	0.021	0.000
	CV%	1.541	0.000	1.928	3.576	1.876	9.778	2.895	2.859	6.282

## Data Availability

The data presented in this study are available on request from the corresponding author.
